# Role of Antenatal 2D Echocardiography (Echo) in Predicting Peripartum Cardiomyopathy in High-Risk Pregnant Women

**DOI:** 10.7759/cureus.108416

**Published:** 2026-05-07

**Authors:** Sarvada Umerjikar, Vasantkumar Rathod, Anjita Kommuneni, Tanuja P Pattankar

**Affiliations:** 1 Obstetrics and Gynecology, Bijapur Lingayat District Educational Association (BLDE) (Deemed to be University), Shri B. M. Patil Medical College Hospital and Research Centre, Vijayapura, IND; 2 Obstetrics and Gynecology, District Hospital, Tumkur, IND; 3 Obstetrics and Gynecology, MGM Medical College, Vashi, Navi Mumbai, Navi Mumbai, IND; 4 Community Medicine, Bijapur Lingayat District Educational Association (BLDE) (Deemed to be University), Shri B. M. Patil Medical College Hospital and Research Centre, Vijayapura, IND

**Keywords:** echocardiography, high-risk pregnancy, left ventricular dysfunction, maternal outcomes, peripartum cardiomyopathy

## Abstract

Background and aim

Peripartum cardiomyopathy (PPCM) is a potentially life-threatening condition, and early diagnosis remains challenging due to overlap with physiological symptoms of pregnancy. Antenatal 2D echocardiography (echo) may aid in early detection, particularly in high-risk women. The present study aimed to evaluate the role of antenatal 2D echo in predicting PPCM in high-risk pregnancies and to assess associated maternal and fetal outcomes.

Methods

This prospective observational study was conducted on 100 high-risk pregnant women with gestational age >32 weeks at a district hospital. All participants underwent antenatal 2D echocardiographic evaluation. PPCM was diagnosed based on left ventricular ejection fraction (LVEF) <45%. Maternal and fetal outcomes were assessed. Statistical analysis was performed using the chi-square test and independent t-test, with p < 0.05 considered significant.

Results

PPCM was identified in 9 (9.0%) participants. Preeclampsia was the most common risk factor (41 (41.0%)) and showed a significant association with PPCM (6 (66.7%) vs 35 (38.5%), p = 0.04). All PPCM cases demonstrated reduced LVEF and left ventricular systolic dysfunction. The mean LVEF was significantly lower in the PPCM group compared to non-PPCM participants (p < 0.001). PPCM was associated with significantly adverse maternal and fetal outcomes, including higher rates of ICU admission (6 (66.7%) vs 8 (8.8%)), maternal mortality (1 (11.1%) vs 0 (0.0%)), NICU admission (4 (44.4%) vs 12 (13.2%)), intrauterine growth restriction (3 (33.3%) vs 10 (11.0%)), and prolonged hospital stay (5 (55.6%) vs 12 (13.2%)).

Conclusions

Antenatal 2D echo is a valuable, noninvasive modality for the early detection of PPCM in high-risk pregnancies. The present study demonstrated a significant association between PPCM and hypertensive disorders of pregnancy, particularly preeclampsia, highlighting the importance of targeted screening. Early echocardiographic identification of left ventricular systolic dysfunction facilitates timely multidisciplinary management and may help reduce adverse maternal and fetal outcomes. These findings support the incorporation of antenatal echocardiographic assessment into the routine evaluation of high-risk pregnant women, especially in resource-limited settings.

## Introduction

Peripartum cardiomyopathy (PPCM) is a rare but potentially life-threatening form of heart failure that occurs toward the end of pregnancy or within the months following delivery [1]. It is characterized by left ventricular systolic dysfunction, typically defined by a left ventricular ejection fraction (LVEF) of <45%, occurring in the absence of any identifiable cause of heart failure and without preexisting structural heart disease [2,3]. Although considered uncommon, PPCM remains a significant contributor to maternal morbidity and mortality worldwide, with a higher reported burden in low- and middle-income settings, including India [4,5]. The condition is associated with considerable adverse outcomes, including heart failure, thromboembolism, and even maternal death, thereby posing a major clinical challenge in obstetric care [6].

The clinical presentation of PPCM often overlaps with normal physiological changes of late pregnancy, such as dyspnea, fatigue, and pedal edema, which frequently leads to underdiagnosis or delayed recognition [7]. This overlap makes early identification particularly challenging, especially in high-risk populations. Several maternal risk factors have been consistently associated with PPCM, including hypertensive disorders of pregnancy, anemia, multiple gestation, obesity, diabetes mellitus, and advanced maternal age [7,8]. The presence of these risk factors further increases the vulnerability of pregnant women to cardiac dysfunction, highlighting the need for vigilant screening and monitoring [9].

Echocardiography plays a central role in the diagnosis and evaluation of PPCM, as it is a noninvasive, widely available, and cost-effective imaging modality that allows real-time assessment of cardiac structure and function [10,11]. It enables early detection of left ventricular systolic dysfunction and helps differentiate PPCM from other causes of heart failure in pregnancy [12]. 2D echo, in particular, has been shown to have high sensitivity in detecting early cardiac changes and may serve as a valuable tool in identifying women at risk before the onset of overt clinical symptoms [10].

Despite its clinical utility, there is limited prospective evidence assessing the role of routine antenatal echocardiographic screening in predicting PPCM among high-risk pregnant women, particularly in resource-limited settings where a substantial burden of disease exists. Early identification of subclinical cardiac dysfunction in such populations could potentially improve maternal and fetal outcomes through timely intervention. Therefore, the present study was undertaken to evaluate the role of antenatal 2D echo in predicting PPCM in high-risk pregnancies and to assess the associated maternal and fetal outcomes.

## Materials and methods

This prospective observational study was conducted in the Department of Obstetrics and Gynecology at a district hospital over a period of six months from January 2024 to June 2024, after obtaining approval from the Institutional Ethics Committee (approval IMSRH/IEC/2023-24/DNB-01). The study included high-risk pregnant women with gestational age greater than 32 weeks who attended the antenatal outpatient department or were admitted to the obstetrics ward during the study period. High-risk pregnancy was defined by the presence of conditions such as preeclampsia, gestational hypertension, gestational diabetes mellitus, obesity, twin gestation, elderly primigravida status, or prior COVID-19 infection [13].

Sample size was calculated using the following formula:

$n = \frac{Z^2 \times p \times q}{d^2},$

where n is the required sample size, Z is 1.96 at 95% CI, p is the estimated proportion, q = 1 − p, and d is the absolute precision. Based on previously reported data by Agarwal et al., the maternal mortality associated with PPCM was 11.7%, and this value was used as the expected proportion (p = 0.117; q = 0.883) [5]. Assuming a precision (d) of 6.3% and a 95% CI, the calculated sample size was approximately 100 participants. Considering feasibility constraints and the predefined study duration, a total of 100 high-risk pregnant women were included in the study.

Pregnant women aged 18 years or above with gestational age greater than 32 weeks and identified as high-risk were included in the study. Women presenting with symptoms suggestive of cardiac dysfunction, such as dyspnea, fatigue, or edema, were included in the study. Women with known preexisting cardiac disease, congenital heart disease, severe anemia (hemoglobin <8 g/dL), those receiving treatment for systemic illnesses, and those unwilling to provide consent were excluded from the study.

After obtaining informed consent, a detailed history was recorded using a predesigned pro forma. All participants underwent a thorough clinical examination along with routine antenatal investigations as per institutional protocol, followed by antenatal 2D echocardiographic evaluation performed by a qualified cardiologist. PPCM was diagnosed based on evidence of left ventricular systolic dysfunction, defined primarily by an LVEF <45% [3,14,15]. The echocardiographic findings were used to categorize participants into PPCM and non-PPCM groups for further analysis.

Maternal outcomes assessed included the need for intensive care unit admission, duration of hospital stay, and maternal mortality. Fetal outcomes included intrauterine growth restriction, need for NICU admission, and other adverse outcomes.

Data were entered and analyzed using IBM SPSS Statistics for Windows, version 26.0 (released 2018; IBM Corp., Armonk, NY, USA). Categorical variables were expressed in N (%) format, while continuous variables were presented as mean ± SD. The chi-square test was used for comparison of categorical variables, and the independent t-test was applied for continuous variables. A p-value of < 0.05 was considered statistically significant.

## Results

A total of 100 high-risk pregnant women were included in the study. The majority of participants were aged 18-25 years (52 (52.0%)), followed by 26-30 years (34 (34.0%)), while 14 (14.0%) were above 30 years. Most participants belonged to rural areas (67 (67.0%)), with 33 (33.0%) from urban settings. Primigravida women constituted the majority (61 (61.0%)), compared to 39 (39.0%) multigravida women. Overall, the study population was predominantly young, rural, and primigravida (Table 1).

**Table 1:** Baseline characteristics of study population (N = 100) Data are presented as n (%). No inferential statistical test was applied, as this table describes baseline characteristics.

Variable	Category	n (%)
Age (years)	18-25	52 (52.0%)
	26-30	34 (34.0%)
	>30	14 (14.0%)
Locality	Rural	67 (67.0%)
	Urban	33 (33.0%)
Gravidity	Primigravida	61 (61.0%)
	Multigravida	39 (39.0%)

Preeclampsia was the most common high-risk factor, observed in 41 (41.0%) participants, making it the predominant contributor in this cohort. This was followed by gestational hypertension in 25 (25.0%) and obesity in 21 (21.0%) cases. Twin gestation and gestational diabetes mellitus were each present in 11 (11.0%) participants, while eclampsia was noted in 7 (7.0%). Elderly primigravida and prior COVID-19 infection were less frequent, seen in 5 (5.0%) and 3 (3.0%) cases, respectively. Multiple risk factors were present in some participants, indicating overlapping risk profiles (Table 2).

**Table 2:** Distribution of high-risk factors among study participants (N = 100) Data are presented as n (%). No statistical test was applied, as multiple responses were possible and the table is descriptive in nature. Multiple risk factors were present in some participants.

Risk factor	n (%)
Preeclampsia	41 (41.0%)
Gestational hypertension	25 (25.0%)
Obesity	21 (21.0%)
Twin gestation	11 (11.0%)
Gestational diabetes mellitus	11 (11.0%)
Eclampsia	7 (7.0%)
Elderly primigravida	5 (5.0%)
Prior COVID-19 infection	3 (3.0%)

PPCM, defined by LVEF <45%, was identified in 9 (9.0%) participants, while 91 (91.0%) had normal left ventricular function (LVEF ≥45%). This reflects a notable occurrence of PPCM among high-risk pregnancies in the study population (Table 3).

**Table 3:** Incidence of PPCM based on LVEF (N = 100) Data are presented as n (%). No statistical test was applied, as this table represents categorical distribution. LVEF, left ventricular ejection fraction; PPCM, peripartum cardiomyopathy

Category	n (%)
PPCM (LVEF <45%)	9 (9.0%)
No PPCM (LVEF ≥45%)	91 (91.0%)

All participants with PPCM demonstrated reduced LVEF (<45%) and left ventricular systolic dysfunction (9 (100.0%)), whereas none of the non-PPCM group showed these findings (0 (0.0%)), with a highly significant association (χ² = 99.99, p < 0.001). The mean LVEF was significantly lower in the PPCM group (38.2 ± 4.5) compared to the non-PPCM group (58.1 ± 5.8), with a highly significant difference (t = 10.43, p < 0.001) (Table 4).

**Table 4:** Echocardiographic findings in study participants Categorical data are presented as n (%), and continuous data as mean ± SD. The chi-square (χ²) test was used for comparison of categorical variables, and the independent Student’s t-test was applied for comparison of continuous variables between groups. ^*^ A p-value of <0.05 was considered statistically significant. LV, left ventricle; LVEF, left ventricular ejection fraction; PPCM, peripartum cardiomyopathy

Parameter	PPCM (n = 9)	No PPCM (n = 91)	Statistical value	p-value
LVEF <45%	9 (100.0%)	0 (0.0%)	χ² = 99.99	<0.001^*^
Mean LVEF (%)	38.2 ± 4.5	58.1 ± 5.8	t = 10.43	<0.001^*^
LV systolic dysfunction	9 (100.0%)	0 (0.0%)	χ² = 99.99	<0.001^*^

Preeclampsia showed a statistically significant association with PPCM, being present in 6 (66.7%) cases compared to 35 (38.5%) in the non-PPCM group (χ² = 4.21, p = 0.04). Other risk factors, including gestational hypertension (3 (33.3%) vs 22 (24.2%)), obesity (3 (33.3%) vs 18 (19.8%)), twin gestation (2 (22.2%) vs 9 (9.9%)), gestational diabetes mellitus (1 (11.1%) vs 10 (11.0%)), and eclampsia (2 (22.2%) vs 5 (5.5%)), were more frequently observed in the PPCM group but did not reach statistical significance. Thus, preeclampsia emerged as the most important associated risk factor for PPCM in this study (Table 5).

**Table 5:** Association of risk factors with PPCM Data are presented as n (%). The chi-square (χ²) test was used to assess the association between categorical variables. ^*^ A p-value of <0.05 was considered statistically significant. PPCM, peripartum cardiomyopathy

Risk factor	PPCM (n = 9)	No PPCM (n = 91)	χ²	p-value
Preeclampsia	6 (66.7%)	35 (38.5%)	4.21	0.04^*^
Gestational hypertension	3 (33.3%)	22 (24.2%)	0.36	0.68
Obesity	3 (33.3%)	18 (19.8%)	0.67	0.72
Twin gestation	2 (22.2%)	9 (9.9%)	1.05	0.61
Gestational diabetes mellitus	1 (11.1%)	10 (11.0%)	0.00	0.89
Eclampsia	2 (22.2%)	5 (5.5%)	2.11	0.21

PPCM was associated with significantly worse maternal and fetal outcomes. ICU admission was markedly higher in the PPCM group (6 (66.7%) vs 8 (8.8%); χ² = 22.48, p < 0.001). Maternal mortality was observed in the PPCM group (1 (11.1%)) compared to none in the non-PPCM group (0 (0.0%)); however, this finding should be interpreted with caution given the very small number of events (n = 1), despite showing statistical significance (χ² = 10.21, p = 0.001). Adverse neonatal outcomes were also significantly increased, including NICU admission (4 (44.4%) vs 12 (13.2%); p = 0.01) and intrauterine growth restriction (3 (33.3%) vs 10 (11.0%); p = 0.05). Additionally, prolonged hospital stay (>5 days) was significantly more common among PPCM patients (5 (55.6%) vs 12 (13.2%); p = 0.002). These findings underscore the substantial adverse impact of PPCM on both maternal and neonatal outcomes (Table 6).

**Table 6:** Maternal and fetal outcomes in relation to PPCM Data are presented as n (%). The chi-square (χ²) test was used for comparison between groups. ^*^ A p-value of <0.05 was considered statistically significant. IUGR, intrauterine growth restriction; PPCM, peripartum cardiomyopathy

Outcome	PPCM (n = 9)	No PPCM (n = 91)	χ²	p-value
ICU admission	6 (66.7%)	8 (8.8%)	22.48	<0.001^*^
Maternal mortality	1 (11.1%)	0 (0.0%)	10.21	0.001^*^
NICU admission	4 (44.4%)	12 (13.2%)	6.52	0.01^*^
IUGR	3 (33.3%)	10 (11.0%)	3.84	0.05
Hospital stay >5 days	5 (55.6%)	12 (13.2%)	10.72	0.002^*^

## Discussion

The present study evaluated the role of antenatal 2D echo as a screening tool for predicting PPCM among high-risk pregnant women. The incidence of PPCM in this cohort was 9 (9.0%), based on reduced LVEF (<45%). This incidence is higher than population-based estimates as reported by Agarwal et al. (one in 1340 to one in 11,000 births), which is expected due to the selective inclusion of high-risk pregnancies, thereby enriching the study population with predisposed individuals [5].

The study population was predominantly young, with a higher proportion of primigravida women. Although advanced maternal age is traditionally considered a risk factor, several studies have demonstrated that PPCM commonly affects younger women in developing countries. Joshi et al. reported a mean age of 26.8 years among PPCM patients, while Binu et al. observed a mean age of 25.5 years, findings comparable to the present study [16,17]. These observations suggest that demographic risk profiles may vary geographically, and younger women in resource-limited settings may still be at considerable risk.

In the present study, preeclampsia was the most prevalent high-risk factor, observed in 41 (41.0%) participants, and showed a statistically significant association with PPCM, being present in 6 (66.7%) cases. This finding is consistent with existing literature, where hypertensive disorders of pregnancy have been strongly associated with PPCM. Khade et al. reported preeclampsia in 66% of PPCM cases, while Bello et al. demonstrated a significant association, attributing it to shared mechanisms such as endothelial dysfunction and antiangiogenic imbalance [18,19]. These findings emphasize the need for heightened cardiac surveillance in women with hypertensive disorders.

Other risk factors, including gestational hypertension, obesity, twin gestation, and gestational diabetes mellitus, were more frequently observed among PPCM cases, although they did not achieve statistical significance. This lack of significance may be attributed to the relatively small number of PPCM cases (9 (9.0%)), limiting statistical power. Nevertheless, these factors have been implicated in previous studies as contributors to increased hemodynamic stress and myocardial dysfunction, particularly in cases of multiple gestations and metabolic disorders [20,21].

Antenatal 2D echo in the present study enabled early identification of left ventricular systolic dysfunction, with all PPCM cases demonstrating reduced LVEF and systolic impairment. Echocardiography remains the cornerstone for diagnosis and risk stratification in PPCM due to its noninvasive nature, availability, and ability to provide real-time functional assessment [22]. Biyyani et al. highlighted its utility in early diagnosis and prognostication, particularly in high-risk populations [23]. The significant difference in mean LVEF between PPCM and non-PPCM groups in the present study further reinforces its diagnostic value.

The present study also demonstrated that PPCM is associated with significantly adverse maternal and fetal outcomes. ICU admission was required in 6 (66.7%) PPCM cases compared to 8 (8.8%) in the non-PPCM group, while maternal mortality was observed only in the PPCM group (1 (11.1%)) compared to none in the non-PPCM group; however, this finding should be interpreted with caution given the very small number of events (n = 1). Adverse neonatal outcomes, including NICU admission (4 (44.4%) vs 12 (13.2%)) and intrauterine growth restriction (3 (33.3%) vs 10 (11.0%)), were also significantly higher. These findings are consistent with previous studies. Singh et al. reported increased maternal mortality in patients with severe left ventricular dysfunction, while Binu et al. demonstrated an association between reduced LVEF and poor maternal outcomes [17,21].

The present study has several strengths, such as focused evaluation of high-risk pregnant women, which facilitated early identification of PPCM using antenatal 2D echo. The use of objective echocardiographic criteria and systematic assessment of both maternal and fetal outcomes enhances the clinical relevance of the findings. The study also highlights the important association between hypertensive disorders, particularly preeclampsia, and PPCM, supporting the role of targeted screening in high-risk populations. However, certain limitations should be considered, such as the relatively small sample size and single-center design. The use of convenience sampling may introduce selection bias, and the inclusion of only late gestation high-risk pregnancies restricts applicability to early pregnancy and low-risk groups. Additionally, postpartum women were not included in the study, despite PPCM commonly manifesting after delivery, which may limit the comprehensive assessment of disease incidence. Additionally, the absence of long-term follow-up precludes assessment of recovery, and advanced echocardiographic parameters were not evaluated, which could have provided further prognostic insights.

## Conclusions

Antenatal 2D echo is a valuable, noninvasive tool for early detection of PPCM in high-risk pregnancies. The present study demonstrates that targeted screening can facilitate timely identification of left ventricular systolic dysfunction, particularly in women with hypertensive disorders such as preeclampsia, which showed a significant association with PPCM. Early diagnosis through echocardiographic evaluation enables prompt multidisciplinary management, thereby potentially reducing the risk of adverse maternal and fetal outcomes. These findings support the incorporation of antenatal echocardiographic assessment in the evaluation of high-risk pregnant women to improve clinical outcomes, especially in resource-limited settings.
